# Prediction of *cis*/*trans *isomerization in proteins using PSI-BLAST profiles and secondary structure information

**DOI:** 10.1186/1471-2105-7-124

**Published:** 2006-03-09

**Authors:** Jiangning Song, Kevin Burrage, Zheng Yuan, Thomas Huber

**Affiliations:** 1 Advanced Computational Modelling Centre, The University of Queensland, Brisbane Qld 4072, Australia; 2 Institute for Molecular Bioscience and ARC Centre in Bioinformatics, The University of Queensland, Brisbane Qld 4072, Australia

## Abstract

**Background:**

The majority of peptide bonds in proteins are found to occur in the *trans *conformation. However, for proline residues, a considerable fraction of Prolyl peptide bonds adopt the *cis *form. Proline *cis*/*trans *isomerization is known to play a critical role in protein folding, splicing, cell signaling and transmembrane active transport. Accurate prediction of proline *cis*/*trans *isomerization in proteins would have many important applications towards the understanding of protein structure and function.

**Results:**

In this paper, we propose a new approach to predict the proline *cis*/*trans *isomerization in proteins using support vector machine (SVM). The preliminary results indicated that using Radial Basis Function (RBF) kernels could lead to better prediction performance than that of polynomial and linear kernel functions. We used single sequence information of different local window sizes, amino acid compositions of different local sequences, multiple sequence alignment obtained from PSI-BLAST and the secondary structure information predicted by PSIPRED. We explored these different sequence encoding schemes in order to investigate their effects on the prediction performance. The training and testing of this approach was performed on a newly enlarged dataset of 2424 non-homologous proteins determined by X-Ray diffraction method using 5-fold cross-validation. Selecting the window size 11 provided the best performance for determining the proline *cis*/*trans *isomerization based on the single amino acid sequence. It was found that using multiple sequence alignments in the form of PSI-BLAST profiles could significantly improve the prediction performance, the prediction accuracy increased from 62.8% with single sequence to 69.8% and Matthews Correlation Coefficient (MCC) improved from 0.26 with single local sequence to 0.40. Furthermore, if coupled with the predicted secondary structure information by PSIPRED, our method yielded a prediction accuracy of 71.5% and MCC of 0.43, 9% and 0.17 higher than the accuracy achieved based on the singe sequence information, respectively.

**Conclusion:**

A new method has been developed to predict the proline *cis*/*trans *isomerization in proteins based on support vector machine, which used the single amino acid sequence with different local window sizes, the amino acid compositions of local sequence flanking centered proline residues, the position-specific scoring matrices (PSSMs) extracted by PSI-BLAST and the predicted secondary structures generated by PSIPRED. The successful application of SVM approach in this study reinforced that SVM is a powerful tool in predicting proline *cis*/*trans *isomerization in proteins and biological sequence analysis.

## Background

It is well known that the planar peptide bonds occur predominantly in the *trans *conformation [1], c*is *peptide bonds occur rarely in proteins in that there exists an energy barrier of approximately 20 kcal/mol between the *trans *and *cis *conformation. However, in the case of Xaa-Pro peptide bond (also called peptidyl prolyl isomerization, where Xaa is any amino acid), the difference in energy is only 0.5 kcal/mol between *trans *and *cis *isomerization, and the energy barrier is about 13 kcal/mol. Thus a considerable proportion (about 4–5%) of Xaa-Pro peptide bonds adopts the *cis *conformation, while only 0.03–0.05% Xaa-nonPro bonds occur in the *cis *form [2-4].

In recent years, there are an increasing number of known protein structures determined which exhibit conformational heterogeneity of one or more prolyl peptide bonds [5]. Proline *cis *peptide bonds bear great biological significance in protein structure and function. The importance of proline *cis*/*trans *isomerization as rate-limiting step in protein folding has been well characterized [6-8], for example, it has been suggested to dominate the folding of the alpha subunit of trp synthase in *E. coli *[9]. The isomerization process of Xaa-Pro peptide bonds can be catalyzed and accelerated by the so-called peptidyl prolyl *cis*/*trans *isomerase [10], which are found to be involved in cell signaling and cell replication, and be implicated in the induction of severe diseases such as cancer, AIDS, Alzheimer's disease and other neurodegenerative disorders [11]. In addition, proline isomerization functions as molecular switch due to its potential ability to control protein activity within the confines of the intrinsic conformational exchange [5].

Since high throughput genome sequence projects are producing a large number of raw sequence data, fast and accurate prediction methods are in great demand to annotate protein structural and functional properties. Towards this point, accurate prediction of proline *cis*/*trans *isomerization in proteins would have many important applications in the study of protein structure prediction and rational molecular design. Numerous studies on the corrections of the proline *cis*/*trans *population and the prolyl puckering have been reported by analyzing different non-redundant datasets of protein X-ray structures [1,4,6,12,13]. The results indicated that there exist a significant correlation between *cis *conformation content and the local amino acid sequences adjacent to proline residues.

More recently, Pahlke *et al *employed different statistical methods like Chou-Fasman parameter calculation and occurrence matrices to analyze the probability of the *cis *and *trans *proline conformation and derived patterns for its possible prediction [14]. Recent study on the conservation of *cis *prolyl bonds showed that *cis *prolyl residues are more often conserved than *trans *prolyl ones in evolutionary related proteins, and the overall protein sequence homology is a stronger indicator for the occurrence of *cis *prolyl residues in contrast to the local sequence motifs [15].

However, most of these studies were merely based on statistical analysis of the neighboring residue occurrences of centered proline, without further systematic prediction of proline *cis*/*trans *isomerization from the primary protein sequence. To the best of our knowledge, the first attempt to predict the peptidyl prolyl *cis*/*trans *isomerization on the basis of the amino acid sequences was done by Frömmel and Preissner [16]. They used six different patterns to correctly assign about 72.7% (176 *cis*-prolyl residues in their relatively small dataset of 242 Xaa-Pro bonds) of known *cis*-prolyl residues, by taking into account the neighboring ± 6 residues centered on proline, as well as their physicochemical properties. Later, support vector machine (SVM) were then introduced to implement this task and achieved 76.7% prediction accuracy by using jack-knife test for the *cis *proline residues, using the single amino acid sequence information encoded by binary bits (0 and 1) as input vector [17]. COPS algorithm was developed to predict the *cis*/*trans *peptide bond isomerization based on the conformation parameters [18], but this method only took advantage of the secondary structure information of amino acid triplets, failing to consider the important amino acid sequence information.

In this paper, we propose a novel method to predict the proline *cis*/*trans *isomerization based on support vector machine, which combined the position-specific scoring matrices (PSSM) extracted from the sequence profiles by PSI-BLAST [19] and the predicted secondary structures generated by PSIPRED program [20], as the SVM input vector in addition to the single amino acid sequence information. Our method has been evaluated on a well-resolved non-homologous dataset by 5-fold cross-validation test and achieved an overall prediction accuracy of 71.5% and Matthews Correlation Coefficient (MCC) values of 0.43 that provided a comparable prediction performance with all the previously reported results.

## Results

### Xaa-Pro *cis *and Xaa-Pro trans peptide bond distribution

**Figure 1:**
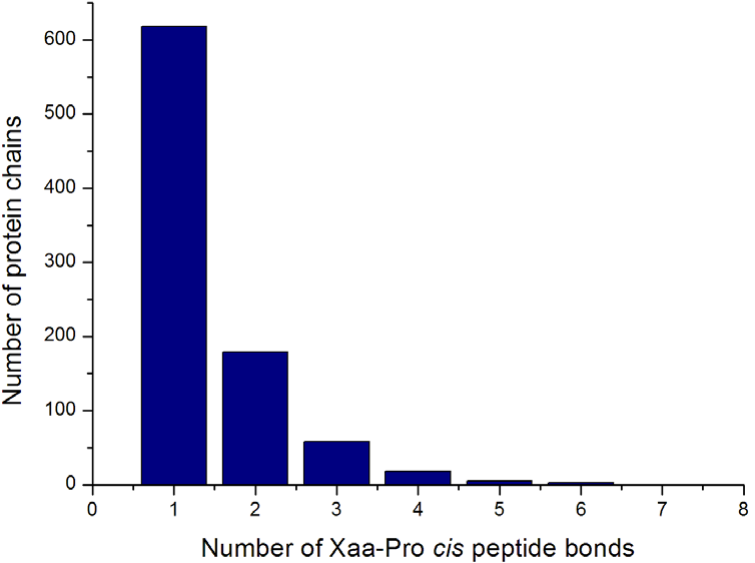
**Distribution of the Xaa-Pro *cis *peptide bonds per protein sequences in the dataset**. Protein chains are grouped according to the number of Xaa-Pro *cis *peptide bonds.

**Figure 2:**
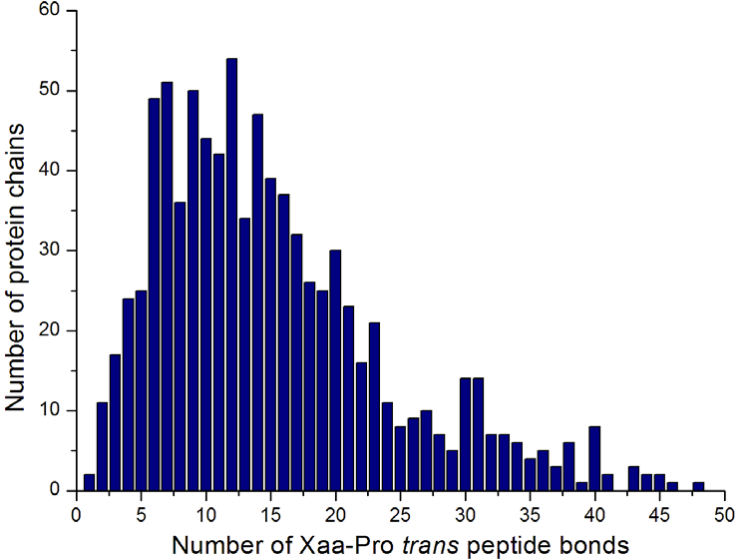
**Distribution of the Xaa-Pro *trans *peptide bonds per protein sequences in the dataset**. Protein chains are grouped according to the number of Xaa-Pro *trans *peptide bonds.

Among the total 2424 protein chains in the current dataset, there are 881 chains containing Xaa-Pro *cis *peptide bonds, in which 1265 prolyl bonds are in *cis *conformation and 12570 are in *trans *form. It was shown that the distribution of Xaa-Pro *cis *peptide bonds is very uneven, and 70% PDB sequences in this dataset have only one prolyl *cis *peptide bond. Less than 3% protein chains have more than three prolyl *cis *bonds (Figure 1). In contrast to the preferably unevenly distributed Xaa-Pro *cis *peptide bonds, the distribution of Xaa-Pro *trans *peptide bonds appears more averagely (Figure 2).

### Effect of different kernel functions and parameters

The selection of the kernel function parameters is an important step for SVM training and testing, because they implicitly determine the structure of the high dimensional feature space when constructing the OSH [40]. Several parameters must be determined in advance to optimize SVM training, such as the regularization parameter C, the γ parameter in RBF kernel, and the *d *parameter in polynomial kernel functions. The parameter C is a regulation parameter which controls the trade-off between margin and the training error.

We used five different SVM models by selecting different combinations of kernel functions and parameters. The prediction accuracy comparison of using different kernel functions and their respective parameters is shown in Table 1. These models are constructed and compared based on single sequence input with window size 11. Model 1 and 2 used single sequence input and second-order and fifth-order polynomial kernel functions, respectively. Model 3, 4 and 5 are all constructed using single sequence input and selecting different choices of C and γ parameters. The results indicate that using RBF kernel could achieve better prediction performance compared with other kernels.

**Figure 3:**
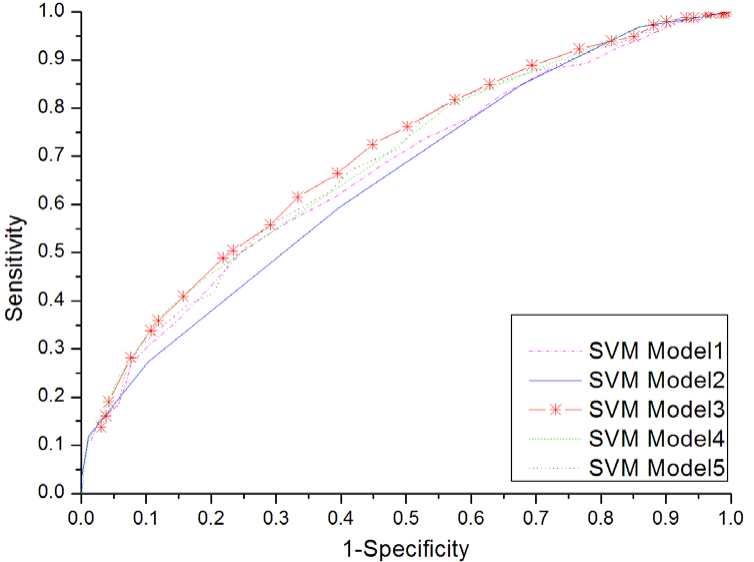
**ROC Curves of five different SVM models**. A ROC curve provides a graphical representation of the relationship between the true-positive and false-positive prediction rate of a SVM model. ROC curve is obtained by plotting all 1-Specificity values (false-positive rate) on the X axis and Sensitivity (true-positive rate) on the Y axis. The resulting area under the ROC curve is an important index for evaluating the classification performance, i.e. the highest and leftmost ROC curve in the plot represents the best SVM model.

As can be seen from the ROC curves in Figure 3, selection of different kernel functions does not make a significant contribution to the final prediction results. Model 3 has the best prediction performance compared with the other models. That means selecting RBF kernel at γ = 0.01 and regularization parameter C = 2.0 could give the better sensitivity values when fixing the specificity values, in comparison with the other SVM models. The results also indicate that using RBF kernel gives a slightly better accuracy than Polynomial kernel, at the cost of longer training and testing time consumed. Therefore in the following analysis, we then selected the mixed combination of RBF kernel at γ = 0.01, C = 2.0 and γ = 0.2, C = 1.0 to evaluate the prediction performance.

### The imbalance problem

The imbalance problem will occur when there is a large difference between the positive and negative samples of the dataset [41]. In this study, the *cis *and *trans *prolyl residues are unbalanced (1265 *cis *prolines versus 27196 *trans *ones). We need to take into consideration this problem because if this proportion is used, the training difficulty will be met and SVM classifier will not converge after the training. And in that case, SVM have a tendency to accurately predict the over-represented class (*trans*) and incorrectly assign the under-represented class (*cis*).

Usually, there are two methods towards overcoming the imbalance problem: (1) Increasing the dataset size of the under-represented samples by random resampling the dataset and (2) Decreasing the size of the over-represented dataset by random removing its samples [41]. Here, we explored the second one. We set the ratio of the size of the positive to negative training samples (the positive-negative-training ratio) at 1:1, since SVM will achieve better accuracy coverage under this ratio.

### Prediction using single sequence information

The SVM has been trained and tested with single sequences encoded as binary bits (0 and 1). In this coding scheme, each amino acid is represented by the 20-dimensional binary vector, e.g. Ala (10000000000000000000), Cys (01000000000000000000), ..., Tyr (00000000000000000001), etc.

Increasing the window size can provide more local sequence information. The window size *w *is defined as the residue numbers involved in the local sequence windows centered on proline, i.e. *w *= 3, 5, 7, 9, 11, 13, 15, 17, 19 in this study. Here, we tried to use different local window sizes to build the SVM models in order to find out which could lead to the best performance. The prediction accuracy is shown in Table 2. The standard deviations of prediction accuracies by 5-fold cross-validation for these variant window sizes are all less than 2%. As expected, the overall prediction accuracy *Q*_2 _(defined in the Methods Section) increases with the enlarging window size and attain its peak at 11. It is understandable since larger window size would have much more noise included while smaller window size would result in less useful information used. Our finding is also consistent with other group's conclusion that more sequence information does not lead to a better prediction [15].

**Figure 4:**
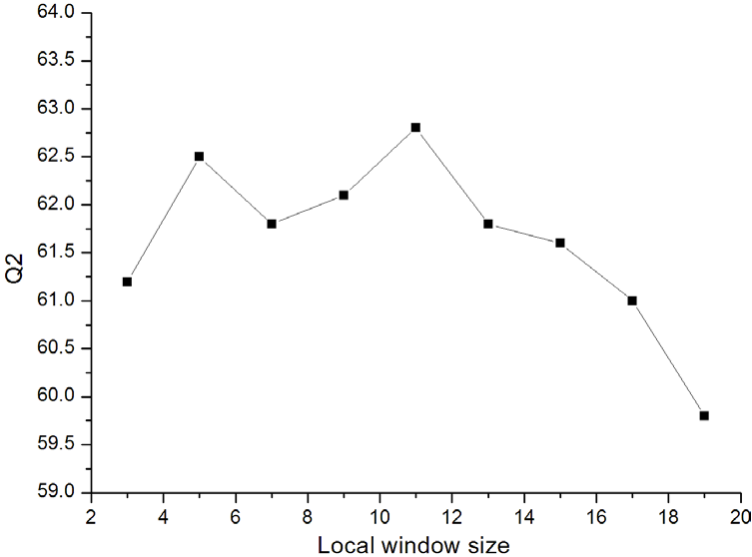
**The prediction accuracy (*Q*_2_) using different local sequence window sizes**. The local window size is defined as the residue numbers involved in the local sequence windows centered on proline.

Accordingly, we then fixed 11 as the optimal window size in the following analysis of this study. Figure 4 is the graphical depiction of the effects of different local sequence window sizes on the prediction accuracy.

### Prediction using amino acid composition of local sequence

We also used the amino acid compositions of different window sizes as SVM input, and compared the influence of different window sizes on the prediction performance. In many cases, amino acid compositions have been proved to result in the improvement of prediction performance to a certain extent. The amino acid composition is calculated by

AA=∑i=120niw
 MathType@MTEF@5@5@+=feaafiart1ev1aaatCvAUfKttLearuWrP9MDH5MBPbIqV92AaeXatLxBI9gBaebbnrfifHhDYfgasaacH8akY=wiFfYdH8Gipec8Eeeu0xXdbba9frFj0=OqFfea0dXdd9vqai=hGuQ8kuc9pgc9s8qqaq=dirpe0xb9q8qiLsFr0=vr0=vr0dc8meaabaqaciaacaGaaeqabaqabeGadaaakeaacqWGbbqqcqWGbbqqcqGH9aqpdaWcaaqaamaaqadabaGaemOBa42aaSbaaSqaaiabdMgaPbqabaaabaGaemyAaKMaeyypa0JaeGymaedabaGaeGOmaiJaeGimaadaniabggHiLdaakeaacqWG3bWDaaaaaa@3B6D@

where *n*_i _is the number of occurrences of amino acid type *i *in the local sequence window of window size *w*.

For this encoding scheme, the input vector of SVM is composed of 20 elements corresponding to the amino acid percentage of twenty residues in the local window sequence. The prediction results based on composition input vectors of different window sizes are listed in Table 3. The prediction performance increases as the window size increases, and reaches the maximum *Q*_2_= 61.6% and MCC = 0.23 at size 15. It is worth noting that the selecting the window size 11 doesn't necessarily result in the best performance in terms of this coding scheme.

The prediction performance reached *Q*_2 _= 61.6% and MCC = 0.23 at the full length. The relatively high accuracy by using only amino acid compositions of the full sequence length mainly comes from the improvement on the Sensitivity value (as high as 72.6%) despite its low Specificity (44.5%), implying that proline *cis*/*trans *isomerization state is also determined by the global sequence information, as well as the local sequence information.

### Prediction using multiple sequence alignment and secondary structure information

In this work, we employed several different encoding schemes, i.e. local sequence ("LS"), amino acid compositions of local sequence ("AA"), multiple sequence alignment in the form of PSI-BLAST profiles ("MS"), predicted secondary structure by PSIPRED ("SS"), and multiple sequence alignment plus secondary structure ("MS+SS"). The prediction results are shown in Table 4.

It is well known that multiple sequence alignment rather than single amino acid sequence could improve the prediction accuracy [25]. In order to further improve the prediction performance, we then included multiple sequence alignment in the form of PSI-BLAST position-specific scoring matrices (PSSMs) as the SVM input. As expected, including evolutionary information in the form of PSI-BLAST profiles could significantly increase the prediction performance. As a result, the MCC improved from 0.26 with single local sequence to 0.40. The considerable improvement in prediction score came from the use of position-specific scoring matrices in the multiple sequence alignment that contained some relevant information of distantly related protein sequences with query proteins [25]. And the PSI-BLAST profiles are represented by the position-specific probabilities of this relevant weighted information, thus greatly enhanced the prediction performance.

Recently, Pahlke *et al *developed a stand alone algorithm COPS to predict the *cis *and *trans *conformation of amino acids in proteins. Their algorithm was based on statistical analysis of the so-called conformation parameters- the extension of Chou-Fasman parameters. COPS derived four rules to predict the *cis *conformation by taking into consideration the secondary structure of amino acid triplets alone [18]. Therefore we wanted to know whether introducing the predicted secondary structure information by PSIPRED as the input to SVM classifier would be contributive or not. As can be seen in Table 4, the overall accuracy *Q*_2 _was 63.6 and the MCC value was 0.27, which was better than that obtained with local sequence ("SS"). The results indicated that including the secondary structure by PSIPRED could provide more useful information for the prediction performance compared with the local sequence alone.

To further improve the prediction performance, we combined the multiple sequence alignment in the form of PSI-BLAST ("MS") and the predicted secondary structure from PSIPRED ("SS"). Among those five SVM models, "MS+SS" provided the best predictions of proline *cis*/*trans *isomerization. For this model, its overall accuracy *Q*_2 _was 71.5% and MCC was 0.43, while the MCC values for "LS", "AA" and "SS" were 0.26, 0.23 and 0.27, respectively. There is also a great improvement in the Sensitivity and Specificity values after using "MS+SS" encoding scheme. The final values of Sensitivity and Specificity are 70.7% and 72.2%, which are 14% and 3.5% higher than that obtained with single sequence alone, respectively. All these prediction scores indicate that using multiple sequence alignment together with the predicted secondary structure considerably increases the number of true positives and true negatives and decreases the over- and under-predictions.

However, our results also showed that simply combining "AA" together with "MS+SS" couldn't result in the better prediction performance than "MS+SS" (data not shown). This may result from the reason that including too many input vectors not only increased the useful information used by SVM classifier but also introduced much noise underlying those vectors at the same time.

**Figure 5:**
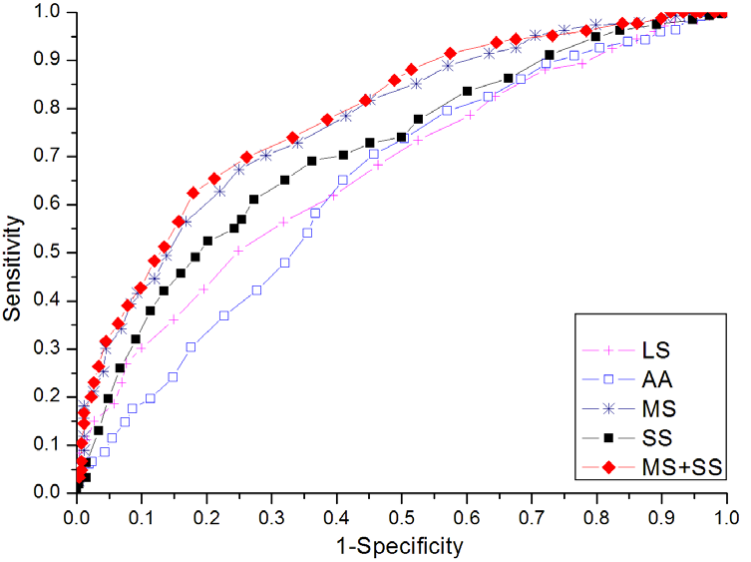
**ROC Curves of five different SVM models**. Five SVM models were constructed using five different sequence encoding schemes: single local sequence ("LS"), amino acid compositions of local sequence ("AA"), multiple sequence alignment ("MS"), secondary structure information ("SS"), and multiple sequence alignment with secondary structure ("MS+SS").

In addition, the performance of different SVM models has also been evaluated by comparing the areas under the receiver operating characteristic (ROC) curves. As can be seen from the ROC Curves in Figure 5, SVM model based on "MS+SS" encoding schemes surpasses all the other models, which means this SVM classifier has better sensitivity values given any choice of specificity compared with other models.

### Comparison with other methods

We need to make an objective comparison among different methods by using their prediction results generated based on the same dataset. In this study, we analyzed the prediction performance of SVM methods, as well as the Naïve Bayes, Logistic regression, *K*-nearest neighbor and decision tree classifiers. The performance comparison of these different classifiers obtained by 5-fold cross-validation is shown in Table 5.

The prediction accuracy of SVM is about 12% and 13% higher than Naïve Bayes and Logistic regression classifiers, respectively. The accuracy difference between SVM classifier and those based on K-nearest neighbor and decision trees are even larger. The same tendency exists for the MCC values. Moreover, the SVM classifier could correctly assign 70.7% of the *cis *proline residues, namely, 13% higher than any other classifier implemented in Weka package used in this study. In contrast, Naïve Bayes and Logistic regression could only recognize about 61% of *trans *proline samples in the dataset, but on the other hand, they failed to predict the *cis *proline ones (less than 60%). Therefore, it is obvious that SVM outperformed other machine learning techniques in implementing the prediction task of proline *cis*/*trans *isomerization based on the same dataset.

There is several works that studied the prediction of prolyl *cis*/*trans *isomerization in the current literature [16-18]. Here, we also made a comparison with those published work, especially the method proposed by Wang *et al *[17], who also used SVM and the same single sequence encoding scheme. The comparison is summarized in Table 6.

Based on the statistical analysis of the neighbors (± 6 residues) of proline residues and their physiochemical properties, Frömmel and Preissner found six patterns that could be applied to assign correctly 72.7% (less than 75%) of known *cis *proline residues [16]. However, it should be pointed out that their result was obtained on a small dataset containing only 242 Xaa-Pro peptide bonds, thus the six patterns found might not be applicable when using larger dataset.

COPS is a stand alone algorithm that was developed based on the extended Chou-Fasman parameters, i.e. the conformation parameters for each amino acid after considering the correlation between the secondary structure information and the *cis*/*trans *conformation [18]. Their prediction was made by using the four rules found, all of which needs to be fulfilled otherwise *trans *would be predicted. As can be seen from Table 6, the prediction accuracy of COPS for the *cis *proline is 63.6% (averaged by 10-fold cross-validation), which is consistent with the result obtained by using SVM based on predicted secondary structure.

Wang *et al *first introduced support vector machine to solve this task and achieved an overall accuracy of 69.8% and 76.7%, when measured by the independence and jack-knife test, respectively. They used the single amino acid sequence information encoded by binary bits (20-dimensional vectors composed of 0 and 1) as the input vector to SVM [17]. Although their prediction accuracy by jack-knife test was better than that of our method, these results were drawn based on a different dataset.

Perhaps we should not attach too much importance to the prediction score here, because it is unfair to compare the different studies using different datasets and accuracy assessment methods. Although different datasets (242 prolyl residues, 2193, 2424 and 8584 proteins) and different prediction performance test methods (self-consistency, jack-knife and n-fold cross-validation) were used, our method achieved a comparable prediction performance, especially after adopting the PSI-BLAST and PSIPRED encoding scheme. Therefore we can conclude that our method was successful in predicting the proline *cis*/*trans *isomerization, with the prediction accuracy at a satisfactory level.

### CISPEPpred web server

The CISPEPpred web server [51] has been developed for the prediction of proline *cis*/*trans *isomerization in proteins by using the method in this work. This server provides two SVM models based on the single sequence and the multiple sequence alignment in the form of PSI-BLAST profiles along with the secondary structure by PSIPRED, respectively. With the protein sequence submitted in FASTA format, the order of proline residues in the sequence and their respective *cis*/*trans *isomerization state predicted will be generated. Additional information including the introduction, methodology and the PDB chain list used in this study can be found at this website.

## Discussion

Prediction of proline *cis*/*trans *isomerization is important in the understanding of protein structure and function. In the present work, we carried out the extensive prediction study of proline *cis*/*trans *isomerization by using different encoding schemes and developed a novel tool to implement this task based on support vector machines. We investigated the effect of different SVM kernel functions and their corresponding parameters and found that using RBF kernel achieved better prediction performance compared with polynomial kernel and linear kernel. Our results indicate that SVM classifier built on multiple sequence alignment in the form of PSI-BLAST profiles could yield better performance, the prediction accuracy improved from 62.8% with single sequence to 69.8%, while MCC improved from 0.26 with single local sequence to 0.40. This result strengthens the fact that introducing multiple sequence alignments could improve the prediction performance rather than single sequence. Moreover, using PSI-BLAST profiles in the form of position-specific scoring matrices contribute significantly to improve the prediction performance together with the predicted secondary structures by PSIPRED, the prediction accuracy was further improved to *Q*_2 _of 71.5% and MCC of 0.43.

There are three important factors that account for the prediction performance of our method. Firstly, we employed SVM in the present study which is a new machine learning method based on Statistical Learning Theory. SVM has many attractive features not only in its fast speed and scalability, but also in its ability to extract and condense information contained in the training samples. Secondly, multiple sequence alignment in the form of PSI-BLAST profiles was used. The PSI-BLAST profiles were generated by searching the remote protein homologs against the NCBI non-redundant database, thus containing the useful evolutionary information [27]. Thirdly, the predicted secondary structure by PSIPRED was also used. Recent studies indicate that the neighboring secondary structure of prolines could be used to predict the *cis*/*trans *conformation and achieved a good performance [14,18]. PSIPRED is considered as one of the best secondary structure prediction methods. The strategy of using multiple sequence alignment in the form of PSI-BLAST profiles together with predicted secondary structure information by PSIPRED has been successfully applied in the prediction of α-turn [25] and β-turn types in proteins [26,27].

Further improving the prediction accuracy with only local sequence information remains a difficult and challenging task, in that peptidyl prolyl *cis*/*trans *isomerization is also determined by its intrinsically flexible properties of *cis*/*trans *switches inside the proline residues themselves, which could in turn increase the prediction difficulty. The prediction performance is related to the global information on the protein level like the amino acid compositions. Moreover, recent study also suggested that global sequence homology is a strong indicator for the occurrence of *cis *prolyl residues [15]. The key point is to find out accurate descriptors of *cis*/*trans *proline residues and put forward appropriate encoding schemes in order to serve efficiently as the classifier input vectors. However, the unbalanced distribution of *cis*/*trans *samples in proteins and the property *cis*/*trans *conformation switch of further increases the difficulty in predicting their states. It should be pointed out that the overall prediction accuracy of *cis*/*trans *isomerization is correlated with the ratio between these two classes, perhaps it would be reasonable for us not to attach much importance to the absolute *Q*_2 _values. In this aspect, MCC could be considered as the coequal measures of the classification performance.

Future improvements may be achieved by combining several available methods and incorporating more possible information to describe the prolyl *cis*/*trans *peptide bonds, for example, protein solvent accessibility. Since protein solvent accessibility is an important factor in determining protein structure and function, including this information might enhance the prediction performance. In fact, recent studies also indicated that *cis *proline residues are more frequently found in surface accessible areas compared to the *trans *prolines [14]. Therefore, further improvement is anticipated to be attained by combining some non-local structural descriptors of proteins such as protein structural classes and homologs and the local sequence profiles of proline residues like protein solvent accessibility profiles. Thus future work is possible to focus on this direction and improve the prediction accuracy by constructing such multiple feature vectors.

## Conclusion

In this paper, we developed a new method to predict the proline *cis*/*trans *isomerization in proteins based on support vector machine. The CISPEPpred web server has been designed to implement this task. The preliminary experiments indicate that using RBF kernels could lead to better prediction performance than that of polynomial and linear kernel functions. We proposed several different sequence encoding schemes and compared their resulting prediction performance. The purpose of this study was to find which kind of information input can lead to the best prediction result. The prediction accuracies were averaged by using 5-fold cross-validation. It was found that using multiple sequence alignments could significantly improve the prediction performance, the prediction accuracy increased from 62.8% with single sequence to 69.8% and MCC from 0.26 to 0.40. Moreover, if coupled with the secondary structure information predicted by PSIPRED, the prediction accuracy was further improved to 71.5% and MCC of 0.43, 9% and 0.17 higher than the accuracy achieved based on the singe sequence information. The successful application of SVM approach in this study reinforced that SVM is a powerful prediction tool for extracting the relationship between proline *cis*/*trans *isomerization and primary amino acid sequence. We believe that CISPEPpred will be a useful tool for proline *cis*/*trans *isomerization prediction and will provide helpful and complementary information in understanding protein structure and function.

## Methods

### Dataset

In the present study, the dataset comprised 2424 non-homologous protein chains, which was obtained from the Culled PDB list provided by PSICES server [21]. This list was generated on October 15, 2005. All structures in this database were determined by X-ray crystallography method with resolution better than 2.0 Å and R-factor less than 0.25. The sequence identity between each pair of sequences was less than 25%. The protein chains with sequence length shorter than 60 amino acids were excluded in our dataset. Every chain contains at least one proline residues. There are totally 609182 residues in this dataset. The protein chain names can be found in Additional file 1. The detailed information of proline *cis*/*trans *peptide records and protein sequences of each protein chain can be found in Additional file 2 and 3.

Although the PDB files do contain the CISPEP records, we can't directly extract these records in that there may exist some errors for such annotations as the bond angles [22,23]. We calculated the *ω *dihedral angle of the CO-NH bond for each proline residue with the preceding amino acid. Bonds with *ω *dihedral angle between -30° and +30° were considered as *cis *peptide bonds, whereas bonds with *ω *dihedral angle between -180° (or +30°) and -30° (or +180°) were assumed to be *trans*. According to this definition, we gained 28461 *ω *dihedral angles for the Xaa-Pro bonds, which included 1265 *cis *and 27196 *trans *prolyl residues.

### Sequence profiles generated by PSI-BLAST

We used a sliding window method to describe the neighboring sequence environments of proline residues, with local window length 2*l*. The local window was centered on the proline residue and the preceding amino acid. Evolutionary information in the form of multiple sequence alignment profiles generated by PSI-BLAST program was included in this window as the input information. The idea of adopting the intermediate PSI-BLAST generated position-specific scoring matrix (PSSM) as direct input was first proposed by Jones [20]. Now this method has been widely used in protein secondary structure prediction [24-27], subcellular localization prediction [28], disulfide connectivity prediction [29], solvent accessibility prediction [30], protein-protein binding site prediction [31], DNA binding site prediction [32], protein B-factor profile [33], as well as protein contact number prediction [34]. Including evolutionary information in the form of PSI-BLAST profiles has been proved to improve the prediction accuracy by a significant increment of about 3–5% in these problems.

Here, we applied this method as the first use of PSSM in proline cis/trans isomerization prediction. Firstly, we obtained the NCBI nr database [35], which contained all known databases: all non-redundant GenBank translations, SwissProt, PIR, PDB, PRF, and NCBI RefSeq database. Then, blastpgp program was run to query each protein in our dataset against the NCBI nr database to generate the PSSM profiles, by three iterations of PSI-BLAST, with a cutoff *E*-value of 0.001. After that, these profiles were scaled to the required 0–1 range by the following standard logistic function

f(x)=11+exp⁡(−x)
 MathType@MTEF@5@5@+=feaafiart1ev1aaatCvAUfKttLearuWrP9MDH5MBPbIqV92AaeXatLxBI9gBaebbnrfifHhDYfgasaacH8akY=wiFfYdH8Gipec8Eeeu0xXdbba9frFj0=OqFfea0dXdd9vqai=hGuQ8kuc9pgc9s8qqaq=dirpe0xb9q8qiLsFr0=vr0=vr0dc8meaabaqaciaacaGaaeqabaqabeGadaaakeaacqWGMbGzcqGGOaakcqWG4baEcqGGPaqkcqGH9aqpdaWcaaqaaiabigdaXaqaaiabigdaXiabgUcaRiGbcwgaLjabcIha4jabcchaWjabcIcaOiabgkHiTiabdIha4jabcMcaPaaaaaa@3D50@

where *x *is the raw profile matrix value. The scaled PSSM profiles were then used as the input information to SVM.

The use of PSSM profiles can avoid the time-consuming multiple sequence alignment procedures. The PSSM is a protein sequence is an *M *× 20 matrix, where *M *is the target sequence length and 20 is the number of amino acid types. Each element of the matrix represents the log-odds score of each amino acid at one position in the multiple alignments. The window size 2*l*+1 indicated the scope of the vicinity of the target prolyl peptide bonds, determining how much neighboring sequence information was included in the prediction. In order to evaluate the influence of different window sizes on the prediction performance, we selected 9 windows sizes to build our SVM predictors, i.e. *M *= 3, 5, 7, 9, 11, 13, 15, 17, 19 (*l = *1, 2, 3, 4, 5, 6, 7, 8, 9, respectively).

### Predicted secondary structure by PSIPRED

The predicted probability matrices of secondary structure states from PSIPRED have also been used in prediction. PSIPRED is a well-known program to predict the protein secondary structure, whose output provides the reliability indices (in 0–1 range) for all the three secondary structure states (helix, strand and coli) for each residue in the protein sequence [20]. We directly extracted the *M *× 3 matrix from the output file of PSIPRED using a sliding window scheme, where *M *is the target sequence length and 3 is the number of secondary structure types.

### Support vector machine

The concept of support vector machine (SVM) was first introduced by Vapnik and his coworkers [36,37]. SVM is a new machine learning method based on Statistical Learning Theory (SLT) and has been extensively used in many kinds of pattern recognition problems, such as microarray data analysis [38], protein secondary structure prediction [39], protein subcellular localization prediction [40,42,43], disulfide connectivity prediction [29] and protein solvent accessibility prediction [44]. The SVM approach usually outperforms other machine learning technologies, including artificial neural networks (ANN), K-nearest neighbor (KNN) methods and Bayesian inference classification. The basic idea of SVM is to transform the samples into a high dimensional feature space and construct an Optimal Separating Hyperplane (OSH) that maximize its distance from the closest training samples. The attractive features of SVM lie in its fast speed and scalability, as well as its ability to extract and condense information contained in the training samples. SVM can not only be used deal with two-class classification but also be extended to multi-class problems. More details description of SVM can be found in Vapnik's publications [36,37].

In the present study, we used SVM_light, an implementation of Vapnik's SVM for support vector classification, regression and pattern recognition [45]. 5-fold cross-validation was used on the dataset of 2, 424 protein sequences to evaluate the prediction efficiency of the current method. The whole dataset were randomly divided into 5 subsets of roughly equal size. In each validation step, one subset was selected for testing, while the rest were used as the training dataset. The selection of the kernel function parameters is an important step for SVM training and testing, because implicitly determine the structure of the high dimensional feature space when constructing the OSH [40]. Several parameters must be determined in advance to optimize SVM training, such as the regularization parameter C, the γ parameter in RBF kernel, and the *d *parameter in polynomial kernel functions.

Here, we adopted the polynomial kernel function and Radial Basis Function (RBF kernel) to construct the SVM classifiers:

K(x→i,x→j)=(x→i·x→j+1)d
 MathType@MTEF@5@5@+=feaafiart1ev1aaatCvAUfKttLearuWrP9MDH5MBPbIqV92AaeXatLxBI9gBaebbnrfifHhDYfgasaacH8akY=wiFfYdH8Gipec8Eeeu0xXdbba9frFj0=OqFfea0dXdd9vqai=hGuQ8kuc9pgc9s8qqaq=dirpe0xb9q8qiLsFr0=vr0=vr0dc8meaabaqaciaacaGaaeqabaqabeGadaaakeaacqWGlbWscqGGOaakcuWG4baEgaWcamaaBaaaleaacqWGPbqAaeqaaOGaeiilaWIafmiEaGNbaSaadaWgaaWcbaGaemOAaOgabeaakiabcMcaPiabg2da9iabcIcaOiqbdIha4zaalaWaaSbaaSqaaiabdMgaPbqabaGccqWIpM+zcuWG4baEgaWcamaaBaaaleaacqWGQbGAaeqaaOGaey4kaSIaeGymaeJaeiykaKYaaWbaaSqabeaacqWGKbazaaaaaa@4549@

K(x→i,x→j)=exp⁡(−r‖x→i−x→j‖2)
 MathType@MTEF@5@5@+=feaafiart1ev1aaatCvAUfKttLearuWrP9MDH5MBPbIqV92AaeXatLxBI9gBaebbnrfifHhDYfgasaacH8akY=wiFfYdH8Gipec8Eeeu0xXdbba9frFj0=OqFfea0dXdd9vqai=hGuQ8kuc9pgc9s8qqaq=dirpe0xb9q8qiLsFr0=vr0=vr0dc8meaabaqaciaacaGaaeqabaqabeGadaaakeaacqWGlbWscqGGOaakcuWG4baEgaWcamaaBaaaleaacqWGPbqAaeqaaOGaeiilaWIafmiEaGNbaSaadaWgaaWcbaGaemOAaOgabeaakiabcMcaPiabg2da9iGbcwgaLjabcIha4jabcchaWjabcIcaOiabgkHiTiabdkhaYnaafmaabaGafmiEaGNbaSaadaWgaaWcbaGaemyAaKgabeaakiabgkHiTiqbdIha4zaalaWaaSbaaSqaaiabdQgaQbqabaaakiaawMa7caGLkWoadaahaaWcbeqaaiabikdaYaaakiabcMcaPaaa@4B54@

where in the case of polynomial kernel, the degree d needs to be tuned, and the γ parameter and the regularization parameter C for RBF kernel need to be regulated.

### Waikato environment for knowledge analysis (Weka)

Weka 3.4.5 is a comprehensive Java library of machine learning package [46] providing an implementation of many state-of-the-art learning and data mining algorithms [47], such as decision trees, rule sets, Bayesian classifiers, support vector machines, logistic and linear regression, multi-layer perceptrons and nearest-neighbor methods, as well as meta-learners like bagging, boosting, stacking, etc [47]. The algorithms provided by Weka can be classified into three types: classification, regression and feature selection. More information about Weka can be found in [48]. In this work, we selected four algorithms to build our classifiers: 1) Naïve Bayes, which is an implementation of the probabilistic Naïve Bayesian classifier; 2) Logistic regression, which is a variation of ordinary regression frequently used when the observed outcome is restricted to two values; 3) lazy IBk, which is based on the k-nearest neighbors classifier that employs the distance metric for classification; 4) J48, which is an implementation of a decision tree learner.

The input data for Weka classifiers is represented in ARFF (attribute-relation function format), consisting of the list of all instances with the values for each instance separated by commas ("yes" for *cis *proline fragments and "no" for *trans *proline fragments). As a result of dataset training and testing, a confusion matrix will be generated showing the number of instances of each class that has been assigned.

### Performance assessment

To evaluate the prediction performance of the classifiers, we used the 5-fold cross-validation method, i.e. the dataset were randomly divided into ten groups, with each group containing roughly equal numbers of protein sequences. Each group was singled out in turn as the testing dataset, while the remaining proteins in other groups were used as the training dataset.

Four different measurements have been used to measure the prediction performance of our method. The sensitivity (*sens*; also called *recall*, i.e. the fraction of positive examples that are predicted correctly) is given by

sensitivity=TPTP+FN
 MathType@MTEF@5@5@+=feaafiart1ev1aaatCvAUfKttLearuWrP9MDH5MBPbIqV92AaeXatLxBI9gBaebbnrfifHhDYfgasaacH8akY=wiFfYdH8Gipec8Eeeu0xXdbba9frFj0=OqFfea0dXdd9vqai=hGuQ8kuc9pgc9s8qqaq=dirpe0xb9q8qiLsFr0=vr0=vr0dc8meaabaqaciaacaGaaeqabaqabeGadaaakeaacqWGZbWCcqWGLbqzcqWGUbGBcqWGZbWCcqWGPbqAcqWG0baDcqWGPbqAcqWG2bGDcqWGPbqAcqWG0baDcqWG5bqEcqGH9aqpdaWcaaqaaiabdsfaujabdcfaqbqaaiabdsfaujabdcfaqjabgUcaRiabdAeagjabd6eaobaaaaa@450B@

where *TP *is the number of the true positives and *FN *is the number of false negatives or under-predictions.

The specificity (*spec*; also called *precision*, i.e. the fraction of negative examples that are predicted correctly) is given by

specificity=TNTN+FP
 MathType@MTEF@5@5@+=feaafiart1ev1aaatCvAUfKttLearuWrP9MDH5MBPbIqV92AaeXatLxBI9gBaebbnrfifHhDYfgasaacH8akY=wiFfYdH8Gipec8Eeeu0xXdbba9frFj0=OqFfea0dXdd9vqai=hGuQ8kuc9pgc9s8qqaq=dirpe0xb9q8qiLsFr0=vr0=vr0dc8meaabaqaciaacaGaaeqabaqabeGadaaakeaacqWGZbWCcqWGWbaCcqWGLbqzcqWGJbWycqWGPbqAcqWGMbGzcqWGPbqAcqWGJbWycqWGPbqAcqWG0baDcqWG5bqEcqGH9aqpdaWcaaqaaiabdsfaujabd6eaobqaaiabdsfaujabd6eaojabgUcaRiabdAeagjabdcfaqbaaaaa@44A9@

where *TN *is the number of true negatives, and *FP *is the number of false positives or over-predictions.

The overall prediction accuracy is given by

overall accuracy Q2=TP+TNTP+TN+FP+FN
 MathType@MTEF@5@5@+=feaafiart1ev1aaatCvAUfKttLearuWrP9MDH5MBPbIqV92AaeXatLxBI9gBaebbnrfifHhDYfgasaacH8akY=wiFfYdH8Gipec8Eeeu0xXdbba9frFj0=OqFfea0dXdd9vqai=hGuQ8kuc9pgc9s8qqaq=dirpe0xb9q8qiLsFr0=vr0=vr0dc8meaabaqaciaacaGaaeqabaqabeGadaaakeaacqWGVbWBcqWG2bGDcqWGLbqzcqWGYbGCcqWGHbqycqWGSbaBcqWGSbaBcqqGGaaicqWGHbqycqWGJbWycqWGJbWycqWG1bqDcqWGYbGCcqWGHbqycqWGJbWycqWG5bqEcqqGGaaicqWGrbqudaWgaaWcbaGaeGOmaidabeaakiabg2da9maalaaabaGaemivaqLaemiuaaLaey4kaSIaemivaqLaemOta4eabaGaemivaqLaemiuaaLaey4kaSIaemivaqLaemOta4Kaey4kaSIaemOrayKaemiuaaLaey4kaSIaemOrayKaemOta4eaaaaa@578A@

The Matthews Correlation Coefficient (MCC) [49] is defined as

MCC=TP×TN−FP×FN(TP+FP)(TP+FN)(TN+FP)(TN+FN)
 MathType@MTEF@5@5@+=feaafiart1ev1aaatCvAUfKttLearuWrP9MDH5MBPbIqV92AaeXatLxBI9gBaebbnrfifHhDYfgasaacH8akY=wiFfYdH8Gipec8Eeeu0xXdbba9frFj0=OqFfea0dXdd9vqai=hGuQ8kuc9pgc9s8qqaq=dirpe0xb9q8qiLsFr0=vr0=vr0dc8meaabaqaciaacaGaaeqabaqabeGadaaakeaacqWGnbqtcqWGdbWqcqWGdbWqcqGH9aqpdaWcaaqaaiabdsfaujabdcfaqjabgEna0kabdsfaujabd6eaojabgkHiTiabdAeagjabdcfaqjabgEna0kabdAeagjabd6eaobqaamaakaaabaGaeiikaGIaemivaqLaemiuaaLaey4kaSIaemOrayKaemiuaaLaeiykaKIaeiikaGIaemivaqLaemiuaaLaey4kaSIaemOrayKaemOta4KaeiykaKIaeiikaGIaemivaqLaemOta4Kaey4kaSIaemOrayKaemiuaaLaeiykaKIaeiikaGIaemivaqLaemOta4Kaey4kaSIaemOrayKaemOta4KaeiykaKcaleqaaaaaaaa@5C01@

The value of MCC is 0 for a random assignment and 1.0 for a perfect prediction. All the results obtained here are from 5-fold cross-validation.

We also measured the classification accuracy by using the Receiver Operating Characteristic (ROC) analysis [50]. ROC is a threshold independent measure and classic method in signal processing technique and has been used in the prediction analysis of protein α-turn, β-turn and B-factor profiles [25,26,33]. For a prediction method, ROC plots classification sensitivity as a function of one minus specificity (1-specificity) for all possible thresholds. The resulting area under the ROC curve is considered as an important index for evaluating the classification performance. That means the highest and leftmost ROC curve in the plot represents the best classification method [33].

## Availability and requirements

The prediction web server CISPEPpred is available at [51].

## Abbreviations

PSSM – Position-Specific Scoring Matrix

SVM – Support Vector Machine

SLT – Statistical Learning Theory

ANN – Artificial Neural Network

KNN – K-Nearest Neighbor

OSH – Optimal Separating Hyperplane

RBF – Radial Basis Function

ARFF – Attribute-Relation Function Format

TP – True Positive

FN – False Negative

TN – True Negative

FP – False Positive

*Q*_2 _– Overall prediction accuracy

MCC – Matthews Correlation Coefficient

ROC – Receiver Operating Characteristic

LS – Local Sequence

AA – Amino Acid composition

MS – Multiple Sequence alignment

SS – Secondary Structure

## Authors' contributions

JS conceived the project, implemented the web prediction system and drafted the manuscript. KB and ZY participated in the system design, supervised the process and provided valuable comments and discussions. TH helped design and implement the web server.

## Supplementary Material

Additional file 1 The PDB codes of 2424 protein chains used in this study. The fifth character in PDB codes represents the peptide chain name and "_" means that it has only one peptide chain. Click here for file

Additional file 2 This file contains the CisPep PDB codes, proline *cis *peptide records, corresponding dihedral angles and protein sequences. Click here for file

Additional file 3 This file contains the TransPep PDB codes and their protein sequences. Click here for file

## Figures and Tables

**Table 1:** Prediction accuracy comparison with different kernel functions and parameters. The results were obtained by 5-fold cross-validation.

SVM model	Kernel function	Parameters	Accuracy (%)
1	Polynomial	α = 1, β = 1, d = 2	59.0
2	Polynomial	α = 1, β = 1, d = 5	60.5
3	RBF	γ = 0.01, C = 2.0	62.8
4	RBF	γ = 0.06, C = 2.0	60.5
5	RBF	γ = 0.2, C = 1.0	62.6

**Table 2:** Predictive performance of SVM based on singe sequence inputs of different local window sizes. More details for prediction accuracy measurement are given in the Methods section. The results were obtained by 5-fold cross-validation.

Window size	Prediction accuracy (%)			
	
	*Q*_2_	MCC	*Sensitivity*	*Specificity*
3	61.2	0.22	64.4	57.9
5	62.5	0.25	63.3	61.6
7	61.8	0.24	61.4	62.3
9	62.1	0.24	61.1	63.2
11	62.8	0.26	56.6	68.7
13	61.7	0.23	59.2	63.8
15	61.6	0.23	55.4	67.6
17	61.0	0.22	56.3	65.6
19	59.8	0.19	55.4	63.9

**Table 3:** Predictive performance of SVM based on amino acid compositions of different local window sizes. More details for prediction accuracy measurement are given in the Methods section. The results were obtained by 5-fold cross-validation.

Window size	Prediction accuracy (%)			
	
	*Q*_2_	MCC	*Sensitivity*	*Specificity*
9	59.9	0.20	62.1	57.9
11	60.6	0.21	60.3	60.9
15	61.6	0.23	59.8	63.2
21	60.4	0.21	50.4	69.9
25	59.5	0.19	56.0	62.7
Full length	59.3	0.18	72.6	44.5

**Table 4:** Comparison of predictive performance of SVM based on different encoding input information. More details for prediction accuracy measurement are given in the Methods section. The results were obtained by 5-fold cross-validation.

Methods	Prediction accuracy (%)			
	
	*Q*_2_	MCC	*Sensitivity*	*Specificity*
LS^a^	62.8	0.26	56.6	68.7
AA^b^	61.6	0.23	59.8	63.2
MS^c^	69.8	0.40	70.5	68.7
SS^d^	63.6	0.27	57.8	69.3
MS+SS^e^	71.5	0.43	70.7	72.2

^a^LS: prediction performance for the local sequence encoding scheme;

^b^AA: prediction performance for the amino acid composition encoding scheme of local sequence;

^c^MS: prediction performance for the multiple sequence alignment encoding scheme in the form of PSI-BLAST profile;

^d^SS: prediction performance for the predicted secondary structure encoding scheme by PSIPRED;

^e^MS+SS: prediction performance for the multiple sequence alignment plus secondary structure encoding scheme.

**Table 5:** Comparison of predictive performance with Naïve Bayes, Logistic regression, IBk and J48 classifier. More details for prediction accuracy measurement are given in the Methods section. The results were obtained by 5-fold cross-validation.

Methods	Prediction accuracy (%)			
	
	*Q*_2_	MCC	*Sensitivity*	*Specificity*
SVM	71.5	0.43	70.7	72.2
Naïve Bayes	59.1	0.18	57.0	61.1
Logistic regression	58.7	0.17	56.6	60.8
IBk (*K*-nearest neighbors)	52.9	0.06	44.9	60.5
J48 (decision trees)	54.2	0.09	53.6	54.7

**Table 6:** Comparison of predictive performance with other methods.

Methods	Prediction accuracy (%)		Dataset used	Prediction performance evaluation method
			
	*Q*_2_	MCC		
Statistical pattern^a^	72.7	-	242 Xaa-Pro bonds	self-consistency
COPS^b^	63.6	-	8584 proteins	10-fold cross-validation
SVM single sequence^c^	69.8	-	2193 proteins	independence test
SVM single sequence^d^	76.6	0.53	2193 proteins	Jack-knife
SVM single sequence^e^	62.8	0.26	2424 proteins	5-fold cross-validation
SVM PSI-BLAST^f^	69.8	0.40	2424 proteins	5-fold cross-validation
SVM PSIPRED^g^	63.6	0.27	2424 proteins	5-fold cross-validation
SVM PSI-BLAST and PSIPRED^h^	71.5	0.43	2424 proteins	5-fold cross-validation

^a^Prediction accuracy reported by Frömmel and Preissner [16]. The result cannot be determined from the paper.

^b^Prediction accuracy estimated based on the average statistical results of COPS [18].

^c^Prediction accuracy using independence test reported by Wang *et al *[17].

^d^Prediction accuracy using jack-knife test reported by Wang *et al *[17].

^e^Prediction accuracy of SVM based on single sequence encoding scheme using our dataset.

^f^Prediction accuracy of SVM based on PSI-BLAST encoding scheme using our dataset.

^g^Prediction accuracy of SVM based on PSIPRED encoding scheme using our dataset.

^h^Prediction accuracy of SVM based on PSI-BLAST and PSIPRED encoding scheme using our dataset.
