# Supplementary material for: Prediction of cis/trans isomerization in proteins using PSI-BLAST profiles and secondary structure information
Source: BMC Bioinformatics. 2006 Mar 9;7:124. doi: 10.1186/1471-2105-7-124 (PMC1450308; doi:10.1186/1471-2105-7-124)
Supplement: Additional file 1 — The PDB codes of 2424 protein chains used in this study. The fifth character in PDB codes represents the peptide chain name and "_" means that it has only one peptide chain. [file 1471-2105-7-124-S1.doc]

The PDB codes of 2424 protein chains used in this study. The fifth character in PDB codes represents the peptide chain name and “_” means that it has only one peptide chain.

| PDB ID | | | | | | | |
| --- | --- | --- | --- | --- | --- | --- | --- |
| 1A12A  1A1X_  1A34A  1A3AA  1A3C_  1A4IA  1A62_  1A6M_  1A6Q_  1A73A  1A76_  1A8D_  1A8L_  1A8O_  1ABA_  1AE9A  1AF7_  1AGQA  1AH7_  1AHO_  1AIL_  1AJSA  1AK0_  1AKO_  1AL3_  1ALU_  1AMF_  1AMUA  1AMX_  1AOCA  1AOL_  1AQUA  1AQZA  1ARB_  1ATG_  1ATZA  1AVWB  1AVYA  1AY7B  1AYOA  1AZO_  1B12A  1B25A  1B2PA  1B3AA  1B4FA  1B4PA  1B65A  1B66A  1B6A_  1B8OA  1B9WA  1BDO_  1BEA_  1BEHA  1BF2_  1BGC_  1BGF_  1BKF_  1BKRA  1BM8_  1BQCA  1BQUA  1BRT_  1BS0A  1BTEA  1BTKA  1BTN_  1BU8A  1BUPA  1BX4A  1BXYA  1BY2_  1BYI_  1BYRA  1C1DA  1C1KA  1C1YB  1C2AA  1C3CA  1C3PA  1C4QA  1C52_  1C5EA  1C75A  1C7KA  1C8UA  1C8XA  1C8ZA  1C96A  1C9OA  1CB8A  1CC8A  1CCWA  1CCWB  1CDY_  1CEWI  1CFB_  1CHD_  1CHMA  1CI4A  1CIPA  1CL8A  1CLC_  1CMCA  1CNV_  1COZA  1CP2A  1CPO_  1CQ3A  1CQMA  1CQQA  1CRUA  1CRZA  1CS0A  1CS1A  1CSN_  1CT5A  1CTF_  1CUJ_  1CUK_  1CV8_  1CVRA  1CXQA  1CY5A  1CZAN  1CZPA  1D02A  1D0CA  1D0DA  1D0QA  1D1QA  1D2NA  1D2OA  1D2SA  1D2TA  1D2VA  1D2VC  1D2ZA  1D2ZB  1D3BA  1D3BB  1D3CA  1D3GA  1D3YA  1D4OA  1D4TA  1D4XG  1D5TA  1D7PM  1D8HA  1D8WA  1D9CA  1DBWA  1DBXA  1DC1A  1DCS_  1DD9A  1DDWA  1DEKA  1DEUA  1DFMA  1DFUP  1DG6A  1DGWX  1DGWY  1DI2A  1DJ0A  1DJ8A  1DK0A  1DK8A  1DKIA  1DLJA  1DLWA  1DMGA  1DMHA  1DNLA  1DOWA  1DOZA  1DP4A  1DP7P  1DPE_  1DPGA  1DQAA  1DQEA  1DQGA  1DQPA  1DQZA  1DS1A  1DSZA  1DTDB  1DUSA  1DUVG  1DVOA  1DWKA  1DXRC  1DY5A  1DYPA  1DZFA  1DZKA  1E0BA  1E0TA  1E19A  1E1HA  1E1HB  1E25A  1E29A  1E2KA  1E2WA  1E30A  1E42A  1E4CP  1E4FT  1E58A  1E5KA  1E6BA  1E6CA  1E6IA  1E6UA  1E7LA  1E85A  1E8CA  1EAJA  1EAQA  1EARA  1EAYC  1EB6A  1EC7A  1EDG_  1EERA  1EERB  1EEXA  1EEXB  1EEXG  1EF8A  1EFDN  1EGWA  1EJ0A  1EJ2A  1EJBA  1EJDA  1EKQA  1EKRA  1EL6A  1ELKA  1ELUA  1EOKA  1EQ2A  1ES5A  1ES9A  1ESWA  1EU1A  1EU3A  1EU8A  1EUVA  1EUWA  1EVLA  1EW4A  1EW6A  1EWFA  1EX2A  1EXTA  1EY4A  1EYBA  1EYEA  1EYHA  1EYQA  1EZ3A  1EZGA  1EZIA  1EZWA  1F00I  1F0XA  1F1EA  1F1MA  1F2LA  1F32A  1F3UA  1F3UB  1F3VA  1F46A  1F5MA  1F5NA  1F60A  1F60B  1F74A  1F7LA  1F86A  1F8EA  1F9VA  1F9YA  1FC3A  1FCQA  1FDR_  1FG7A  1FI2A  1FIPA  1FIT_  1FIUA  1FJ2A  1FJJA  1FK5A  1FKMA | 1FL0A  1FLMA  1FM0D  1FM0E  1FN9A  1FNLA  1FO8A  1FP2A  1FP3A  1FPOA  1FR2A  1FR2B  1FS1B  1FS7A  1FSGA  1FT5A  1FTRA  1FVIA  1FVKA  1FX2A  1FXOA  1FY7A  1FYEA  1G12A  1G1KA  1G2BA  1G2RA  1G3KA  1G3P_  1G4MA  1G5AA  1G5HA  1G5TA  1G61A  1G66A  1G6GA  1G6SA  1G73A  1G8EA  1G8KA  1G8MA  1G8QA  1G9GA  1GA6A  1GA8A  1GAI_  1GAKA  1GBS_  1GCA_  1GCI_  1GCQC  1GD0A  1GD2E  1GDEA  1GHEA  1GK9A  1GK9B  1GKMA  1GKPA  1GL4A  1GMUA  1GMXA  1GNLA  1GNUA  1GNYA  1GO3E  1GO3F  1GOTB  1GOTG  1GP0A  1GP1A  1GP6A  1GPIA  1GPPA  1GPR_  1GPUA  1GQ8A  1GQEA  1GQIA  1GQZA  1GS5A  1GS9A  1GSA_  1GSOA  1GTKA  1GTTA  1GTVA  1GU2A  1GU4A  1GU7A  1GUDA  1GUIA  1GUQA  1GUXA  1GUXB  1GV9A  1GVEA  1GVFA  1GVJA  1GVP_  1GWEA  1GWMA  1GWUA  1GWYA  1GX5A  1GXJA  1GXMA  1GXQA  1GXRA  1GXUA  1GXYA  1GY7A  1GYXA  1H03P  1H05A  1H0HB  1H16A  1H1DA  1H1NA  1H2CA  1H2WA  1H32A  1H4AX  1H4GA  1H4PA  1H4RA  1H4XA  1H5UA  1H65A  1H6FA  1H6KA  1H6LA  1H70A  1H72C  1H7CA  1H7SA  1H80A  1H8EA  1H8ED  1H8EG  1H8EH  1H8PA  1H97A  1H99A  1HBKA  1HBNA  1HBNB  1HBNC  1HD2A  1HDHA  1HDOA  1HE1A  1HF8A  1HFES  1HFUA  1HJSA  1HM9A  1HN0A  1HNJA  1HP1A  1HQ0A  1HQ1A  1HQSA  1HRUA  1HS6A  1HT6A  1HUW_  1HW1A  1HX0A  1HX1B  1HX6A  1HXIA  1HXN_  1HXRA  1HYOA  1HYP_  1HZ4A  1HZTA  1I0DA  1I0RA  1I0VA  1I12A  1I19A  1I1JA  1I1QA  1I1WA  1I24A  1I27A  1I2AA  1I2KA  1I2TA  1I36A  1I39A  1I3CA  1I40A  1I4JA  1I4MA  1I4UA  1I52A  1I58A  1I60A  1I6LA  1I71A  1I7NA  1I7WB  1I88A  1I8AA  1I8DA  1IA9A  1IAB_  1IAPA  1IB2A  1IDPA  1IE9A  1IFRA  1IG3A  1IGQA  1IIRA  1IITA  1IJBA  1IJQA  1IJYA  1IKOP  1IKPA  1IN6A  1INLA  1IO0A  1IO1A  1IOMA  1IOW_  1IQ4A  1IQ6A  1IQCA  1IQZA  1IRDB  1IRQA  1IS3A  1ISPA  1ISUA  1IT2A  1ITUA  1ITVA  1ITWA  1ITXA  1IU8A  1IUAA  1IUQA  1IV3A  1IV8A  1IVNA  1IW0A  1IWLA  1IWMA  1IX9A  1IXH_  1IXKA  1IZCA  1IZMA  1J0PA  1J1NA  1J1TA  1J24A  1J27A  1J2LA  1J2RA  1J30A  1J31A  1J3AA  1J3WA  1J4AA  1J54A  1J5PA  1J5UA  1J5WA  1J75A  1J77A  1J79A  1J7DA  1J7XA  1J8BA  1J8RA  1J98A  1J9LA  1JAKA  1JAYA  1JB3A  1JB7B  1JBEA  1JBOA  1JC4A  1JDHA  1JDW_  1JE5A  1JETA  1JF4A  1JF8A  1JFBA  1JFLA  1JFRA  1JFXA  1JG1A  1JH6A  1JHFA | 1JHGA  1JHJA  1JHSA  1JI1A  1JI7A  1JIDA  1JIWI  1JIXA  1JKEA  1JKXA  1JL0A  1JL1A  1JM1A  1JMKC  1JMVA  1JNDA  1JNIA  1JNRA  1JO0A  1JOSA  1JOVA  1JPZA  1JQ5A  1JR2A  1JR7A  1JR8A  1JSDA  1JSDB  1JTGB  1JU2A  1JU3A  1JUBA  1JUVA  1JV1A  1JW9B  1JX4A  1JX6A  1JY1A  1JYAA  1JYEA  1JYHA  1JYKA  1JYOA  1JYSA  1JZ8A  1JZTA  1K04A  1K07A  1K0MA  1K12A  1K1EA  1K2XA  1K2XB  1K3IA  1K3SA  1K3XA  1K3YA  1K4IA  1K4NA  1K55A  1K5CA  1K5NA  1K6KA  1K77A  1K7CA  1K7JA  1K7WA  1K87A  1K8KC  1K8KD  1K8KE  1K8KF  1K8WA  1K92A  1KA1A  1KAEA  1KCMA  1KCQA  1KGDA  1KHBA  1KHCA  1KHXA  1KHYA  1KICA  1KJLA  1KJQA  1KKOA  1KLLA  1KM4A  1KMJA  1KMOA  1KMTA  1KMVA  1KNGA  1KNMA  1KOE_  1KOLA  1KPF_  1KPGA  1KPTA  1KQ6A  1KQFA  1KQFC  1KQPA  1KR4A  1KR7A  1KS8A  1KS9A  1KSHB  1KSOA  1KT6A  1KUGA  1KV7A  1KVEA  1KVEB  1KW3B  1KW4A  1KWFA  1KWGA  1KXOA  1KYFA  1KZFA  1KZQA  1L1DA  1L1LA  1L2HA  1L3KA  1L3LA  1L3PA  1L5OA  1L6KA  1L6PA  1L6RA  1L7AA  1L7LA  1L8AA  1L8BA  1L9LA  1L9XA  1LAM_  1LB3A  1LB6A  1LBU_  1LBVA  1LC0A  1LC5A  1LCI_  1LDDA  1LFPA  1LFWA  1LG7A  1LH0A  1LK2B  1LK9A  1LKI_  1LKKA  1LL2A  1LLFA  1LLMC  1LM5A  1LM8V  1LMIA  1LML_  1LNIA  1LO7A  1LP9E  1LQ9A  1LQTA  1LQVA  1LR5A  1LRIA  1LS1A  1LSHA  1LSHB  1LSLA  1LST_  1LTZA  1LU4A  1LUCA  1LUGA  1LUQA  1LUZA  1LV7A  1LWBA  1LWDA  1LXJA  1LYQA  1LYVA  1LZLA  1M0DA  1M0KA  1M0WA  1M15A  1M1FA  1M1HA  1M1NA  1M1NB  1M1QA  1M22A  1M2DA  1M33A  1M3QA  1M40A  1M48A  1M4IA  1M4JA  1M4LA  1M4TA  1M4VA  1M55A  1M5Q1  1M5WA  1M65A  1M6PA  1M6SA  1M7GA  1M7YA  1M93B  1M9XC  1M9ZA  1MAI_  1MBMA  1MC2A  1MDC_  1MDL_  1MDOA  1ME4A  1MEXL  1MF7A  1MFMA  1MG4A  1MG7A  1MGTA  1MI8A  1MIXA  1MJ4A  1MJ5A  1MK0A  1MK4A  1MKAA  1MKKA  1MLA_  1MML_  1MN8A  1MNAA  1MNNA  1MOLA  1MOQ_  1MPGA  1MQOA  1MSC_  1MSK_  1MTPA  1MTYB  1MTYD  1MTYG  1MTZA  1MUGA  1MUN_  1MUSA  1MUWA  1MVFD  1MVLA  1MW9X  1MWPA  1MWQA  1MXIA  1MXRA  1MY7A  1MZBA  1MZGA  1N08A  1N0QA  1N12A  1N13B  1N1BA  1N1FA  1N1JA  1N2AA  1N2EA  1N2SA  1N2ZA  1N3LA  1N40A  1N4WA  1N55A  1N57A  1N5UA  1N62A  1N62B  1N62C  1N6AA  1N7HA  1N7ZA  1N83A  1N8KA  1N8VA  1N93X  1N97A  1N9EA  1N9LA  1N9PA  1NA3A | 1NAR_  1NBCA  1NBUA  1NC5A  1NE2A  1NE9A  1NEPA  1NF9A  1NFP_  1NG6A  1NH0A  1NH2C  1NH2D  1NI9A  1NIGA  1NIJA  1NJHA  1NJRA  1NKIA  1NKPA  1NKR_  1NLFA  1NLNA  1NLQA  1NLS_  1NNFA  1NNHA  1NNLA  1NNWA  1NO5A  1NOFA  1NOGA  1NOX_  1NPK_  1NPSA  1NPYA  1NQEA  1NQJA  1NR0A  1NRIA  1NRJA  1NRZA  1NSJ_  1NSZA  1NTHA  1NTVA  1NTYA  1NU0A  1NU4A  1NUUA  1NUYA  1NVMB  1NWAA  1NWWA  1NWZA  1NXMA  1NXUA  1NY1A  1NYCA  1NYTA  1NZ0A  1NZJA  1NZNA  1NZYA  1O08A  1O0SA  1O13A  1O1YA  1O20A  1O22A  1O26A  1O2DA  1O3UA  1O3YA  1O4VA  1O4WA  1O4YA  1O54A  1O66A  1O6AA  1O6DA  1O6SB  1O6VA  1O75A  1O7IA  1O7JA  1O7QA  1O82A  1O8BA  1O8XA  1O97D  1O98A  1O9GA  1O9IA  1O9RA  1O9WA  1OA8A  1OAA_  1OB8A  1OBDA  1OBFO  1OBOA  1OCYA  1OD3A  1OD6A  1ODMA  1ODZA  1OE4A  1OERA  1OEWA  1OEYA  1OF8A  1OFCX  1OFLA  1OGDA  1OGIA  1OGOX  1OGQA  1OGSA  1OH0A  1OI0A  1OI2A  1OI7A  1OIHA  1OIS_  1OJ8A  1OJHA  1OJQA  1OJRA  1OK0A  1OK7A  1OM1A  1OMHA  1OMRA  1ON2A  1ON3A  1ONEA  1ONRA  1ONWA  1OO0A  1OO0B  1OOEA  1OOHA  1OOYA  1OPD_  1OQ1A  1OQJA  1OQVA  1OR0B  1OR7A  1ORC_  1ORSC  1ORUA  1ORVA  1OS6A  1OSPO  1OSYA  1OTFA  1OTKA  1OU8A  1OUWA  1OVNA  1OW1A  1OW4A  1OWLA  1OX0A  1OXDA  1OXJA  1OXXK  1OYGA  1OYJA  1OYWA  1OZ2A  1OZ9A  1P0HA  1P0ZA  1P1JA  1P1MA  1P1XA  1P3CA  1P3DA  1P42A  1P4CA  1P4OA  1P57A  1P5DX  1P5FA  1P5VB  1P5ZB  1P7TA  1P90A  1P99A  1P9AG  1P9HA  1PA1A  1PA7A  1PB7A  1PBJA  1PBWA  1PBYA  1PBYB  1PBYC  1PCFA  1PDO_  1PE9A  1PFVA  1PG4A  1PG6A  1PGS_  1PI1A  1PINA  1PJCA  1PJXA  1PK6A  1PKHA  1PKOA  1PL3A  1PLC_  1PMHX  1PMI_  1PMMA  1PN9A  1PO5A  1POC_  1POT_  1PP0A  1PPRM  1PQ1A  1PQ4A  1PQ7A  1PQHA  1PRXA  1PSRA  1PSWA  1PSZA  1PTMA  1PU5A  1PU6A  1PUC_  1PV5A  1PVGA  1PVMA  1PWBA  1PX5A  1PZ4A  1PZ7A  1PZWA  1PZXA  1Q08A  1Q0PA  1Q0RA  1Q0UA  1Q16A  1Q1FA  1Q2HA  1Q2WA  1Q33A  1Q35A  1Q40B  1Q5YA  1Q5ZA  1Q6OA  1Q6ZA  1Q74A  1Q7EA  1Q7FA  1Q7LA  1Q7LB  1Q7ZA  1Q8BA  1Q8DA  1Q8FA  1Q8IA  1Q8RA  1Q92A  1Q9UA  1QA7A  1QAUA  1QAZA  1QB5D  1QB7A  1QCSA  1QD1A  1QDDA  1QF5A  1QF8A  1QFTA  1QG8A  1QGEE  1QGIA  1QH5A  1QHDA  1QJ4A  1QJPA  1QKRA  1QL0A  1QLMA  1QLWA  1QMGA  1QMYA  1QNAA  1QNRA  1QO7A | 1QOPB  1QOYA  1QQFA  1QQP1  1QQP2  1QQP3  1QQP4  1QQQA  1QR0A  1QREA  1QS1A  1QSGA  1QTNA  1QTNB  1QTWA  1QU9A  1QV9A  1QW2A  1QW9A  1QWGA  1QWNA  1QWOA  1QWYA  1QX4A  1QXMA  1QXYA  1QZ5A  1QZ9A  1QZMA  1R0DA  1R0MA  1R0UA  1R0VA  1R12A  1R17A  1R1MA  1R1TA  1R26A  1R29A  1R2MA  1R2QA  1R3JC  1R4PA  1R4VA  1R4XA  1R5LA  1R5MA  1R5RA  1R5YA  1R6DA  1R6JA  1R6LA  1R6WA  1R6XA  1R75A  1R77A  1R7AA  1R7JA  1R7LA  1R89A  1R9FA  1R9LA  1R9WA  1RA0A  1RCQA  1RDQE  1REGX  1REWC  1RFEA  1RFYA  1RG8A  1RGXA  1RGZA  1RHS_  1RI6A  1RIFA  1RIYA  1RJ1A  1RJCA  1RJDA  1RJOA  1RK6A  1RKIA  1RKQA  1RKTA  1RKUA  1RL0A  1RLIA  1RLJA  1RLMA  1RMG_  1RMWA  1RO0A  1RO7A  1ROCA  1RQ2A  1RQBA  1RQJA  1RQPA  1RQWA  1RSGA  1RSS_  1RT8A  1RTQA  1RTTA  1RU4A  1RUTX  1RV9A  1RW1A  1RW7A  1RWHA  1RWIA  1RWJA  1RWRA  1RWZA  1RX0A  1RXDA  1RXQA  1RY9A  1RYAA  1RYIA  1RYLA  1RYOA  1RYQA  1RZ2A  1RZHH  1RZHM  1S12A  1S14A  1S1DA  1S21A  1S29A  1S2KA  1S2OA  1S2XA  1S3CA  1S3EA  1S4BP  1S4KA  1S5AA  1S5DA  1S5UA  1S7IA  1S7KA  1S7ZA  1S95A  1S99A  1S9RA  1S9UA  1SA3A  1SACA  1SAUA  1SBP_  1SBXA  1SBYA  1SBZA  1SDIA  1SDWA  1SE8A  1SEIA  1SENA  1SFLA  1SFP_  1SFSA  1SFXA  1SG0A  1SG4A  1SG6A  1SGJA  1SGMA  1SGWA  1SH8A  1SHEA  1SI6X  1SJWA  1SJYA  1SKZ_  1SLUA  1SMBA  1SMXA  1SNYA  1SO7A  1SQ9A  1SQEA  1SQSA  1SQWA  1SR4B  1SR4C  1SR8A  1SR9A  1SRA_  1SRVA  1SSQA  1SSXA  1STMA  1SU8A  1SUR_  1SUUA  1SVB_  1SVFA  1SVMA  1SVPA  1SW5A  1SX5A  1SX7A  1SXRA  1SYYA  1SZ7A  1SZHA  1SZOA  1SZWA  1T06A  1T0BA  1T0FA  1T0HA  1T0HB  1T0PB  1T0TV  1T15A  1T1DA  1T1JA  1T1UA  1T1VA  1T2DA  1T2WA  1T3TA  1T3YA  1T4BA  1T4FM  1T56A  1T5BA  1T5HX  1T5IA  1T5RA  1T61A  1T6CA  1T6EX  1T6SA  1T6T1  1T6UA  1T7LA  1T7RA  1T82A  1T8KA  1T8TA  1T92A  1T9FA  1T9HA  1T9IA  1TA8A  1TAFA  1TBFA  1TC5A  1TCA_  1TDZA  1TE5A  1TEN_  1TFE_  1TFJA  1TFZA  1TG0A  1TG7A  1TH7A  1THFD  1THQA  1TIF_  1TIG_  1TIQA  1TJOA  1TJXA  1TJYA  1TKEA  1TL2A  1TL9A  1TN6A  1TN6B  1TOVA  1TP5A  1TP6A  1TQ5A  1TQGA  1TQHA  1TQJA  1TR0A  1TR9A  1TS9A  1TT8A  1TU1A  1TU9A  1TUAA  1TUKA  1TUOA  1TUVA  1TUWA  1TVGA  1TVXA  1TW6A  1TWDA  1TWIA  1TWUA  1TWYA  1TX4A  1TXGA | 1TXJA  1TXLA  1TXOA  1TXQA  1TXQB  1TYJA  1TYV_  1TZJA  1TZPA  1TZVA  1TZYA  1TZYB  1TZYC  1TZYD  1TZZA  1U00A  1U02A  1U07A  1U09A  1U0FA  1U0SA  1U14A  1U24A  1U2HA  1U2KA  1U4BA  1U4GA  1U55A  1U5DA  1U5KA  1U5PA  1U5UA  1U5XA  1U60A  1U6DX  1U7GA  1U7IA  1U7KA  1U7LA  1U7PA  1U84A  1U8VA  1U9LA  1UA4A  1UAIA  1UALA  1UANA  1UASA  1UBKL  1UBKS  1UC7A  1UC8A  1UCDA  1UCRA  1UCSA  1UD9A  1UEBA  1UEHA  1UEKA  1UFIA  1UFOA  1UFYA  1UG6A  1UGIA  1UGPA  1UGPB  1UHKA  1UI0A  1UIXA  1UJ2A  1UJ8A  1UJCA  1UJPA  1UK8A  1UKFA  1UKKA  1UM0A  1UMGA  1UMHA  1UMMA  1UMWA  1UNNC  1UNQA  1UOZA  1UPKA  1UPQA  1UPSA  1UPTB  1UQ5A  1UQTA  1URSA  1US0A  1US5A  1USCA  1USGA  1USLA  1USMA  1UT1A  1UT7A  1UTEA  1UTG_  1UUJA  1UUYA  1UUZA  1UV4A  1UV7A  1UVJA  1UW1A  1UW4A  1UW4B  1UWCA  1UWFA  1UWKA  1UWWA  1UWZA  1UX6A  1UXAA  1UXY_  1UYLA  1UZBA  1UZEA  1UZXA  1V05A  1V0AA  1V0EA  1V0WA  1V2BA  1V2XA  1V2ZA  1V30A  1V33A  1V3EA  1V4AA  1V4PA  1V4VA  1V54A  1V54B  1V54C  1V54D  1V54E  1V54F  1V54G  1V54H  1V54I  1V58A  1V5EA  1V5IB  1V5VA  1V6PA  1V6SA  1V70A  1V71A  1V73A  1V74A  1V74B  1V77A  1V7BA  1V7LA  1V7RA  1V7WA  1V7ZA  1V84A  1V8HA  1V96A  1V9FA  1V9MA  1V9YA  1VAJA  1VBIA  1VBKA  1VBWA  1VC4A  1VCC_  1VCHA  1VCTA  1VD5A  1VD6A  1VDKA  1VE1A  1VE2A  1VE4A  1VEFA  1VETA  1VFJA  1VFLA  1VFYA  1VGGA  1VGJA  1VGYA  1VH4A  1VH5A  1VHH_  1VHNA  1VHSA  1VHTA  1VHUA  1VHVA  1VHWA  1VHYA  1VI4A  1VI6A  1VI9A  1VIAA  1VICA  1VIE_  1VIMA  1VIN_  1VIOA  1VIZA  1VJ2A  1VJJA  1VJLA  1VJOA  1VJPA  1VJUA  1VJVA  1VK1A  1VK4A  1VK5A  1VKBA  1VKEA  1VKFA  1VKHA  1VKIA  1VKKA  1VKMA  1VKNA  1VKPA  1VKWA  1VKYA  1VL1A  1VL7A  1VLAA  1VLPA  1VLRA  1VLS_  1VLYA  1VM9A  1VMBA  1VMEA  1VMHA  1VNS_  1VP4A  1VP6A  1VPBA  1VPDA  1VPJA  1VPMA  1VPRA  1VPSA  1VPT_  1VQ3A  1VQQA  1VQSA  1VQUA  1VQZA  1VR5A  1VR7A  1VR8A  1VR9A  1VRAA  1VRAB  1VRMA  1VSRA  1VYBA  1VYIA  1VYKA  1VYRA  1VYYA  1VZIA  1VZWA  1VZYA  1W07A  1W0HA  1W0NA  1W0PA  1W1HA  1W1OA  1W23A  1W2FA  1W2WA  1W2WB  1W2YA  1W3OA  1W41A  1W44A  1W4RA  1W4SA  1W4XA  1W50A  1W53A  1W5QA  1W5RA  1W6SA  1W6SB  1W78A  1W79A  1W8KA  1W8SA  1W94A  1W96A  1W99A  1W9GA | 1W9HA  1WA5B  1WA5C  1WB4A  1WC3A  1WCV1  1WCWA  1WD3A  1WDCA  1WDCC  1WDDA  1WDDS  1WDJA  1WDPA  1WDVA  1WEHA  1WER_  1WHI_  1WHO_  1WHSA  1WHSB  1WHZA  1WIWA  1WJ9A  1WJXA  1WK8A  1WKAA  1WKCA  1WKQA  1WL8A  1WL9A  1WLEA  1WLGA  1WLJA  1WLUA  1WLYA  1WLZA  1WM3A  1WMHA  1WMHB  1WMWA  1WMXA  1WN2A  1WNAA  1WO8A  1WOCA  1WOHA  1WOJA  1WOQA  1WOUA  1WOYA  1WOZA  1WP5A  1WPAA  1WPBA  1WPCA  1WPNA  1WPOA  1WPUA  1WQAA  1WQJB  1WQWA  1WRDA  1WRRA  1WS0A  1WS8A  1WTEA  1WTJA  1WU4A  1WU9A  1WUBA  1WURA  1WV9A  1WVFA  1WVGA  1WW7A  1WWIA  1WWJA  1WXXA  1WYBA  1WZ3A  1WZ8A  1WZUA  1X0LA  1X13A  1X2IA  1X6IA  1X6OA  1X6ZA  1X74A  1X7DA  1X7YA  1X7YB  1X82A  1X8BA  1X8DA  1X8QA  1X8VA  1X91A  1X99A  1X9DA  1X9IA  1XAKA  1XAUA  1XB3A  1XBIA  1XCRA  1XD3A  1XDFA  1XDNA  1XDZA  1XE1A  1XE7A  1XEOA  1XEWX  1XEWY  1XFFA  1XFIA  1XFKA  1XFSA  1XG0A  1XG0C  1XG4A  1XGKA  1XGWA  1XH9A  1XIWA  1XIWB  1XIZA  1XJ4A  1XJEA  1XJUA  1XJVA  1XKFA  1XKIA  1XKPA  1XKPB  1XKPC  1XKRA  1XKWA  1XKYA  1XLQA  1XLYA  1XM3A  1XMKA  1XMTA  1XO0A  1XOCA  1XODA  1XPMA  1XPPA  1XQ6A  1XQAA  1XQOA  1XRKA  1XRUA  1XS0A  1XSQA  1XSVA  1XSZA  1XT5A  1XTLA  1XTPA  1XTTA  1XU9A  1XUBA  1XUUA  1XV2A  1XV5A  1XVHA  1XVWA  1XW3A  1XW8A  1XWTA  1XWVA  1XY7A  1XYIA  1XZOA  1XZZA  1Y08A  1Y0BA  1Y0HA  1Y0KA  1Y0NA  1Y0UA  1Y0YA  1Y12A  1Y1PA  1Y2QA  1Y42X  1Y4WA  1Y5IC  1Y60A  1Y6XA  1Y6ZA  1Y71A  1Y7BA  1Y7PA  1Y7RA  1Y7TA  1Y7YA  1Y80A  1Y81A  1Y88A  1Y8AA  1Y93A  1Y96A  1Y96B  1Y9IA  1Y9LA  1Y9QA  1Y9WA  1YACA  1YARA  1YARO  1YB0A  1YB3A  1YBZA  1YC5A  1YC9A  1YCDA  1YD0A  1YD9A  1YDIA  1YDYA  1YE8A  1YFQA  1YFUA  1YG9A  1YGE_  1YGTA  1YH3A  1YIIA  1YJ1A  1YJ7A  1YJSA  1YKDA  1YKJA  1YKSA  1YLEA  1YLHA  1YLKA  1YLLA  1YLMA  1YLXA  1YM3A  1YMTA  1YN3A  1YN9A  1YNBA  1YNDA  1YNFA  1YO1A  1YO3A  1YOCA  1YOZA  1YPQA  1YPYA  1YQ2A  1YQ5A  1YQEA  1YQGA  1YQHA  1YQSA  1YRCA  1YRKA  1YROB  1YSBA  1YSQA  1YSRA  1YT3A  1YT8A  1YTLA  1YTVM  1YU0A  1YU2A  1YU4A  1YUKA  1YUKB  1YV5A  1YVIA  1YVRA  1YW4A  1YWMA  1YX1A  1YXCA  1YXYA  1YY6A  1YYDA  1Z0PA  1Z0WA  1Z15A  1Z2NX  1Z2UA  1Z2WA  1Z3EA  1Z3XA  1Z45A  1Z4RA  1Z53A  1Z5OA  1Z5ZA  1Z67A | 1Z6MA  1Z6NA  1Z6OA  1Z70X  1Z72A  1Z77A  1Z7AA  1Z84A  1Z8KA  1Z90A  1Z9LA  1ZA0A  1ZAIA  1ZARA  1ZAVA  1ZB1A  1ZC3B  1ZCEA  1ZCKA  1ZD0A  1ZDYA  1ZE3C  1ZE3D  1ZEDA  1ZEQX  1ZESA  1ZGKA  1ZGMA  1ZHAA  1ZHHB  1ZHMA  1ZHSA  1ZHVA  1ZHXA  1ZI8A  1ZIN_  1ZJCA  1ZK4A  1ZKEA  1ZKKA  1ZKOA  1ZL0A  1ZLDA  1ZLHB  1ZLQA  1ZMAA  1ZMTA  1ZN6A  1ZP4A  1ZPDA  1ZPSA  1ZR6A  1ZRN_  1ZRSA  1ZS4A  1ZS7A  1ZS9A  1ZT3A  1ZTDA  1ZUOA  1ZV1A  1ZVTA  1ZVZA  1ZWXA  1ZX0A  1ZX8A  1ZXIB  1ZXIC  1ZXIF  1ZXUA  1ZY7A  1ZZKA  1ZZLA  1ZZMA  256BA  2A0B_  2A0KA  2A10A  2A13A  2A14A  2A15A  2A1HA  2A1IA  2A2CA  2A2MA  2A35A  2A3NA  2A50B  2A5LA  2A61A  2A65A  2A67A  2A6CA  2A6SA  2A72A  2A7BA  2A7MA  2A8AA  2A8KA  2A8NA  2A97A  2A9DA  2A9IA  2A9KA  2A9KB  2A9SA  2AB0A  2ABBA  2ABK_  2ADOA  2AE0X  2AEBA  2AEUA  2AFWA  2AGDA  2AGTA  2AH5A  2AI4A  2AJ6A  2AK5A  2AKAA  2AKAB  2ALIA  2AMDA  2AMHA  2AN1A  2ANPA  2ANWA  2ANYA  2AO0A  2AO9A  2AOBA  2AORA  2AP1A  2AP3A  2APJA  2APQA  2AQ6A  2AQJA  2AQWA  2ARCA  2ARZA  2AS0A  2AS9A  2ASBA  2ASFA  2ATZA  2AUWA  2AV4A  2AVDA  2AWDA  2AWGA  2AWPA  2AX6A  2AX8A  2AX9A  2AXOA  2AXYA  2AYH_  2AYVA  2B0AA  2B0MA  2B0VA  2B1XA  2B1XB  2B2HA  2B3GA  2B49A  2B4GA  2B4WA  2B4ZA  2B6EA  2B6HA  2BAA_  2BBKH  2BBKL  2BEMA  2BF5A  2BG1A  2BG5A  2BGIA  2BH4X  2BHUA  2BI0A  2BIBA  2BIQA  2BJ0A  2BJ4A  2BJFA  2BJIA  2BJNA  2BJQA  2BJUA  2BJVA  2BK9A  2BKRA  2BKRB  2BKXA  2BKYA  2BL1A  2BL8A  2BL9A  2BLNA  2BLRA  2BM5A  2BMOA  2BMOB  2BNJA  2BO4A  2BO9B  2BOGX  2BOPA  2BPTA  2BR9A  2BREA  2BRFA  2BSHA  2BSJA  2BSWA  2BSYA  2BT9A  2BV2A  2BV9A  2BVFA  2BVPA  2BW3A  2BW4A  2BYCA  2BYWA  2BZ1A  2BZGA  2BZLA  2BZSA  2BZVA  2C0AA  2C0CA  2C0NA  2C1DA  2C1DB  2C1GA  2C1IA  2COVD  2CY7A  2D28C  2D2JA  2D2YA  2DNJA  2DPMA  2ENG_  2FDX_  2GDM_  2HDDA  2HFT_  2HRVA  2IGD_  2ILK_  2LISA  2MCM_  2MHR_  2NACA  2NLRA  2PGD_  2POR_  2PSPA  2PTD_  2PTH_  2PVBA  2SAK_  2SCPA  2SICI  2TGI_  2TNFA  2TPSA  3BAMA  3CHBD  3CLA_  3DAAA  3EIPA  3EZMA  3FAPB  3GCB_  3HTSB  3LZT_  3NUL_  3PCGA  3PROC  3PVIA  3SEB_  3SIL_  3TDT_  3THIA  3VUB_  4BCL_  4EUGA  4MT2_  4UBPA  4UBPB  5CSMA  7A3HA  7AHLA  7HBIA  7ODCA  8ABP_ |
